# Tumours modulate the systemic vascular response to anti‐angiogenic therapy

**DOI:** 10.1002/jat.4301

**Published:** 2022-03-02

**Authors:** Adam Hargreaves, Simon T. Barry, Alison Bigley, Jane Kendrew, Shirley Price

**Affiliations:** ^1^ PathCelerate Ltd. Macclesfield UK; ^2^ Bioscience, Early Oncology AstraZeneca Cambridge UK; ^3^ OracleBio Ltd., BioCity Scotland North Lanarkshire UK; ^4^ Sygnature Discovery Ltd. Macclesfield UK; ^5^ Faculty of Health and Medical Sciences University of Surrey Guildford UK

**Keywords:** anti‐angiogenic, autotaxin‐lysophosphatic acid signaling, Calu‐6, endocrine, fibroblast growth factor, tumour–host, vascular modulation, vascular toxicity, xenograft

## Abstract

Toxicologic evaluation of new drug candidates routinely utilizes healthy animals. In oncology, there remains a limited understanding of the effects of novel test candidates in a diseased host. For vascular modulating agents (VMAs), an increased understanding of preclinical tumour–host interaction, and its potential to exacerbate or alleviate ‘off‐target’ effects of anti‐angiogenic administration, could aid in the prediction of adverse clinical outcomes in a defined cancer patient. We have previously reported that the implantation and growth of a range of human‐ and mouse‐derived tumours leads to structural vascular and, potentially, functional signalling changes within host mouse endocrine tissues, indicating possible roles for tumour‐ and host‐derived cytokines/growth factors and the liberation of myeloid‐derived suppressor cells in this phenomenon. Here, we further demonstrate that the growth of the Calu‐6 xenograft is associated with a resistance to VMA‐induced mouse peripheral endocrine vascular rarefaction (toxicity), with potential functional impact, notably with respect to mixed tyrosine kinase inhibition. The pathogenesis of these findings indicates a potential role for both tumour‐ and host‐derived basic fibroblast growth factor (bFGF), with associated upregulation in the intra‐tumoural autotaxin‐lysophosphatic acid signalling axis.

## Abstract 2

The growth of the Calu‐6 xenograft is associated with a resistance to vascular modulating agent‐induced mouse peripheral endocrine vascular rarefaction (toxicity), with potential functional impact, notably with respect to mixed tyrosine kinase inhibition. The pathogenesis of these findings indicates a potential role for basic fibroblast growth factor, with associated upregulation in the autotaxin‐lysophosphatic acid signalling axis.

## 1 INTRODUCTION

Over the last 20 years, the advancement of clinical anti‐angiogenic therapy has heralded varying success in the treatment of a wide range of tumours. Both monotherapeutic and combinatory approaches have been utilized, to overcome various forms of treatment resistance and to increase drug efficacy. However, despite notable positive achievements, patient side effects, variably predicted from preclinical animal testing, have impacted the clinical utility of a number of these vascular‐targeted drugs (Brinda et al., 2016; Chen & Cleck, 2009; Hanna et al., 2020; Hayman et al., 2012; Izzedine et al., 2009; Kamba et al., 2006; Kamba & McDonald, 2007; Pena‐Hernandez et al., 2019; Syrigos et al., 2011; Touyz et al., 2018; Yang et al., 2013; Zhang et al., 2016).

As the toxicologic investigation of drug candidates on the developmental pathway is routinely tested in healthy animals (Kim & Sharpless, 2012; Morgan et al., 2017, 2013), there remains a limited understanding of the effects of vascular modulating agents (VMAs) in a diseased preclinical setting. An increased understanding of preclinical tumour–host interaction, and its potential to exacerbate or alleviate ‘off‐target’ effects of anti‐angiogenic administration, could aid in the prediction of adverse clinical outcomes in a defined cancer patient.

During preliminary investigations concerning the development of novel structural biomarkers of vascular modulation (Hargreaves et al., 2017), we determined that the transplantation and growth of a Calu‐6 xenograft tumour could potentially attenuate systemic vascular toxicities associated with anti‐angiogenic administration (Figure S1).

We further recently demonstrated that the implantation and growth of a range of human‐ and mouse‐derived tumours leads to structural vascular and, potentially, functional signalling changes within host mouse endocrine tissues, indicating possible roles for tumour‐ and host‐derived cytokines/growth factors, and the liberation of myeloid‐derived suppressor cells (MDSCs) in this phenomenon (Hargreaves et al., 2021).

This work sought to evaluate these observations, with respect to their potential effect upon, and/or association with, the induction of VMA‐induced peripheral endocrine vascular rarefaction (toxicity) in the mouse.

## 2 MATERIALS AND METHODS

### 2.1 VMA treatment studies

To assess the repeatability of initial studies incorporating the Calu‐6 tumour, and to determine if this response was specific to M396647‐induced mixed TKI (Figure S1), or if it had more general applicability, two additional treatments (one at varying dosage) were investigated (Table 1).

**TABLE 1:** Study groups used to assess the effects of various VMA treatment regimens upon the development of systemic endocrine vascular toxicity, in the presence or absence of a tumour burden

Group (*n* = 6 per group)	Control/treated	Route of dosing	Treatment/dose
Non‐tumour‐bearing *nu/nu*	Control	Oral	Vehicle
Non‐tumour‐bearing *nu/nu*	Control	i.p.	Vehicle
Non‐tumour‐bearing *nu/nu*	Treated	Oral	AZ10167514 6 mg/kg/day (efficacious dose)
Non‐tumour‐bearing *nu/nu*	Treated	Oral	AZ10167514 12 mg/kg/day (maximum tolerated dose)
Non‐tumour‐bearing *nu/nu*	Treated	i.p.	DC101 15 mg/kg/day
Calu‐6 tumour‐bearing *nu/nu*	Control	Oral	Vehicle
Calu‐6 tumour‐bearing *nu/nu*	Control	i.p.	Vehicle
Calu‐6 tumour‐bearing *nu/nu*	Treated	Oral	AZ10167514 6 mg/kg/day (efficacious dose)
Calu‐6 tumour‐bearing *nu/nu*	Treated	Oral	AZ10167514 12 mg/kg/day (maximum tolerated dose)
Calu‐6 tumour‐bearing *nu/nu*	Treated	i.p.	DC101 15 mg/kg/day

*Note*: The duration of dosing for all groups was 28 days.

Abbreviations: i.p., intra‐peritoneal; VMA, vascular modulating agent.

All female athymic nude (Swiss *nu/nu* genotype) mice were supplied by the Rodent Breeding Unit (AstraZeneca, Alderley Park, UK) and housed in negative pressure isolators with 12‐h light/dark cycles and provided with sterilized food (supplied by Special Diet Services, Alderley Park, UK) and water ad libitum. The mice weighed ∼25 g and were at least 8 weeks of age at study commencement. Animals were randomized into either vehicle control non‐tumour‐bearing, vehicle control tumour‐bearing, test article‐treated non‐tumour‐bearing or test article‐treated tumour‐bearing groups (*n* = 6 per group).

Calu‐6 (human lung anaplastic carcinoma) xenografts were established on the flank of female athymic nude (Swiss *nu/nu* genotype) mice by subcutaneous injection of 1 × 10^6^ cells in Matrigel TM (BD Biosciences, New Jersey, USA). When tumours reached a volume of 0.1–0.7 cm^3^ (10–14 days after the graft; Table S1), mice were randomized into the various vehicle control/treatment groups, prior to dosing. Tumour sizes were assessed twice weekly by bilateral Vernier calliper measurement. Length was calculated as the longest diameter across the tumour, with width as the corresponding perpendicular measure. Tumour volume was calculated using the formula (length × width) × √(length × width) × (π/6).

### 2.2 Test articles

For the in vivo work, the test compound AZ10167514 (AstraZeneca, Alderley Park, UK) was suspended in a 1% (v/v) solution of polyoxyethylene (20) sorbitan mono‐oleate in deionised water vehicle. AZ10167514 is a mixed vascular endothelial growth factor receptor (VEGFR) tyrosine kinase inhibitor (TKI), and a structurally similar analogue to AZD2171 (Cediranib), with potent inhibition of the tyrosine kinase of VEGFR‐2, and additional inhibition of VEGFR‐3, c‐Kit and platelet‐derived growth factor receptor alpha and beta (PDGFR‐α, PDGFR‐β) (Wedge et al., 2005).

Dosing of AZ10167514 was via oral gavage, once daily, at a dosage of either 6 mg/kg/day (established efficacious dose), or 12 mg/kg/day (established maximum tolerated dose), for a period of 28 days. Mice within a corresponding oral gavage vehicle control group were administered vehicle in isolation.

DC101 (Cell Essentials Inc., Massachusetts, USA) is a mouse‐specific monoclonal IgG, the administration of which leads to neutralization/blockage of VEGFR‐2 (Bocci et al., 2004).

DC101 administration was via intra‐peritoneal (i.p.) injection, once daily, at a dosage of 15 mg/kg/day, for a period of 28 days. Mice within a corresponding i.p. vehicle control group were administered phosphate‐buffered saline (PBS) vehicle in isolation (0.4 ml).

The incorporation of the above dosing regimens intended to explore the effects of differential pharmacology, the influence of low versus high‐dose and the variability of an extended period of treatment, over and above that examined during preliminary investigations.

Following study completion, all mice were culled by terminal narcosis with 5:1 CO_2_/O_2_ mixture, followed by exsanguination.

All procedures were conducted in accordance with Home Office (UK) and local ethical review committee guidelines and complied with the Animals Scientific Procedures Act 1986. Animals were monitored throughout for body weight variations and clinical signs indicative of systemic toxicity/morbidity. Tumours were also measured to ensure that the mean diameter of growth did not exceed 10 mm and that necrosis‐associated skin breakdown or exudation did not arise.

### 2.3 Tissue harvesting, processing and vascular/cell quantification

Following the *in life* phase, selected endocrine organs (right adrenal gland, thyroid gland and pancreas) were removed from all animals (the adrenal gland weighed). Tissues were fixed, embedded and processed for CD31 immunohistochemical (IHC) staining, light microscopic analysis and 3D *Fibrelength Density* analysis, using the Definiens XD image analysis platform (version 2.0.4 Definiens AG), with Tissue Studio and Developer XD, as described previously (Hargreaves et al., 2017). Additional sections of the tissues were stained with haematoxylin and eosin (H&E) for the detection of any morphologic abnormality.

### 2.4 Quantitation of potential endocrine signaling changes in the circulation

To evaluate the endocrinological response of the mouse host to the presence of tumour burden, and the response to varying treatments, serum samples for adrenocorticotrophic hormone (ACTH) and thyroid stimulating hormone (TSH) were quantified using the Milliplex mouse pituitary/thyroid magnetic bead panel (MPTMAG‐49K; EMD Millipore, Massachusetts, USA).

In addition, to assess the induction of pro‐angiogenic signalling, a series of xenograft (human; angiopoietin‐2, endothelin‐1, fibroblast growth factor (FGF)‐1, FGF‐2, granulocyte colony stimulating factor (G‐CSF), interleukin‐8 (IL‐8), leptin, placental growth factor (PlGF) and VEGF‐A) or host (mouse; endothelin‐1, FGF‐2, G‐CSF, IL‐6, leptin, PlGF‐2, prolactin, tumour necrosis factor alpha [TNFa] and VEGF‐A)‐derived cytokines/growth factors were quantified using Milliplex MAP kits (HAGP1MAG‐12K and MAGPMAG‐24K; EMD Millipore, Massachusetts, USA), on a Luminex 200™ System, using xPONENT software (Luminex Corporation, Texas, USA).

Whole blood samples were taken from the tail vein prior to study termination. Samples were placed in a covered test tube, allowing the blood to clot at room temperature for 30 min. Clot separation was undertaken by centrifuging the samples at 10,000 rpm, for 10 min at 4°C, in a refrigerated centrifuge (Eppendorf, Thermo Fisher Scientific, Massachusetts, USA). The serum was then placed into clean polypropylene tubes using a Pasteur pipette, before immediate analysis, as per the manufacturer's guidelines (PROTOCOL_FOR_00003798MAN_MPTMAG‐49K; PROTOCOL_HAGP1MAG‐12K; PROTCOL_MAGPMAG‐24K; Merck Millipore, Massachusetts, USA).

### 2.5 Fluorescence‐activated cell sorting (FACS) bone marrow analysis

In order to analyse distinct lineages of bone marrow‐derived cells, a combination of the differential expression of leukocyte common antigen (CD45, MCA103F; Bio‐Rad Serotec, Oxfordshire, UK) and transferrin receptor (CD71, MCA1033PE; Bio‐Rad Serotec, Oxfordshire, UK) was coupled with side scatter analysis and the DNA stain LDS‐751 (Molecular Probes, L‐7595; Thermo Fisher Scientific, Massachusetts, USA), the latter separating immature versus mature red cells. For further analysis of endothelial precursor cells (EPCs), phycoerythrin (PE)‐conjugated CD202b (Biolegend, California, USA) monoclonal antibodies (5 μl) were additionally incorporated into the protocol.

Fresh bone marrow suspensions were prepared by obtaining femoral samples from all animals at termination, cleaning residual tissue with a scalpel and removing the diaphyseal portion. A 27‐gauge needle on a 3‐ml syringe was used to place 2 ml of PBS +50% fetal bovine serum (FBS) through the bone and into a 12 × 75‐mm tube. The expelled solution was drawn up through the bone and back into the syringe; the process repeated 5 times. The cell suspension was filtered through a 100‐μm nylon mesh filter before underlaying with 1‐ml FBS, centrifuging at 3000 rpm for 5 min at 4°C (Eppendorf, Thermo Fisher Scientific, Massachusetts, USA) and resuspending the pellet in 4 ml of PBS +0.5% bovine serum albumin (BSA).

Fluorescein isothiocyanate (FITC)‐conjugated CD45 (5 μl) and PE‐conjugated CD71 (10 μl) monoclonal antibodies (Bio‐Rad Serotec, Oxfordshire, UK) were added to 100 μl of the bone marrow cell suspension and incubated on ice in the dark for 20 min. Cells were washed with PBS containing 0.5% BSA and re‐centrifuged at 3000 rpm for 5 min at 4°C. The resulting cell pellet was resuspended in 0.5‐ml PBS +0.5% BSA, plus 20 μl of LDS‐751 staining solution (Molecular Probes, L‐7595; Thermo Fisher Scientific, Massachusetts, USA). Samples were then placed in the dark for 20 min, prior to flow cytometric analysis, incorporating negative control samples (adding only 10‐μl PBS/BSA; 5‐μl CD45:FITC; 10‐μl CD71:PE; and 20‐μl LDS‐751, to 4 wells, respectively, before analogous processing). Analysis was performed using a Becton Dickinson FACSCanto II, with FACSDiva software (BD Biosciences, New Jersey, USA), as per the manufacturer's instructions.

### 2.6 Reverse transcription polymerase chain reaction (PCR)

To assess the relative levels of intra‐tumoural gene expression, ribonucleic acid (RNA) was isolated from tumour fragments using the RNeasy Mini Kit (Qiagen, Hilden, Germany), according to the manufacturer's instructions. On‐column DNase digestion was performed using the RNase‐free DNase Kit (Qiagen, Hilden, Germany). RNA concentration was measured using the NanoDrop ND1000 (Thermo Fisher Scientific, Massachusetts, USA).

For fluidigm profiling, mouse specific TaqMan Gene Expression Assays were designed and supplied by Life Technologies (Thermo Fisher Scientific, Massachusetts, USA). Reverse transcription was performed with 50 ng of total RNA in a final volume of 20 μl, using the High Capacity Complementary Deoxyribonucleic Acid (cDNA) Reverse Transcription Kit (Thermo Fisher Scientific, Massachusetts, USA). The following thermal profile was used in the reaction: 25°C for 10 min; 37°C for 120 min; 85°C for 5 s; and 4°C for 2 min. Pre‐amplification was performed with 1.25 μl of resulting cDNA in a final volume of 5 μl, using a pool of TaqMan assays at a final dilution of 1 in 100 and TaqMan PreAmp Master Mix (Thermo Fisher Scientific, Massachusetts, USA). The following thermal profile was used in the reaction: 95°C for 10 min; 14 cycles of 95°C for 15 s; and 60°C for 4 min. Pre‐amplified samples were diluted 1:5 with Tris/Ethylenediaminetetraacetic acid buffer, prior to analysis on the Fluidigm BioMark System (Fluidigm, California, USA). Sample and assay preparations for 96.96 Fluidigm Dynamic Arrays (DA) were performed according to the manufacturer's instructions. Briefly, samples were mixed with DA Sample Loading Reagent (Fluidigm, California, USA) and TaqMan Gene Expression Master Mix (Applied Biosystems, Massachusetts, USA). Assays were mixed with DA Assay Loading Reagent (Fluidigm, California, USA). The 96.96 Fluidigm Dynamic Arrays were primed and loaded on a Controller (Fluidigm, California, USA), and quantitative PCR (qPCR) was performed on a BioMark System (Fluidigm, California, USA), using the following thermal profile: 50°C for 2 min; 70°C for 30 min; 25°C for 10 min; 50°C for 2 min; 95°C for 10 min; 40 cycles of 95°C for 15 s; and 60°C for 1 min. Data were analysed using the Fluidigm BioMark Real‐Time PCR Analysis software, version 3.1.3 (Fluidigm, California, USA), and gene expression values were calculated using the Delta C(T) method.

### 2.7 Western blot

To confirm the findings as observed by 3D *Fibrelength Density* analysis, in terms of protein expression, the left adrenal gland of each test animal was snap frozen at necropsy and transferred to liquid nitrogen, before thawing in radioimmunoprecipitation assay buffer solution containing cOmplete*™* Protease Inhibitor Cocktail and Phosphatase Inhibitor Cocktail (Merck Millipore, Massachusetts, USA). Sonication was performed for 20 s and followed by centrifugation at 5000 rpm, for 10 min at, 4°C, in a refrigerated centrifuge (Eppendorf, Thermo Fisher Scientific, Massachusetts, USA). Supernatant was harvested, and the protein concentration determined using the Thermo Scientific Pierce Bicinchoninic Acid Protein Assay Kit (Thermo Fisher Scientific, Massachusetts, USA); 100‐μg cell extracts were resolved in 10% sodium dodecyl (lauryl) sulphate‐polyacrylamide gels. These were electrophoretically transferred onto a polyvinylidene difluoride membrane (Millipore, Massachusetts, USA). Western blot analysis was performed for total CD31 and VEGFR‐2, using the following antibodies: CD31 (cat. no.124432, rabbit polyclonal Ig [Abcam, Cambridge, UK]) and VEGFR‐2 (cat. no. 39256, rabbit polyclonal Ig [Abcam, Cambridge, UK]). Anti‐glyceraldehyde 3‐phosphate dehydrogenase antibody (GAPDH); cat. no. sc‐32233, mouse monoclonal Ig (Santa Cruz Biotechnology, Texas, USA) was used to verify equal protein loading and transfer. Densitometric analysis was undertaken using the LI‐COR® Image Studio Lite version 3.1 (LI‐COR Biosciences, Nebraska, USA). The relative intensities of CD31 and VEGFR‐2 expression were quantified relative to the protein loading control (GAPDH).

### 2.8 Statistics

For statistical analysis, data were examined for normal distribution using the Shapiro–Wilk test. All comparisons containing normally distributed data groups were subject to unpaired *t*‐test analysis with Welch's correction (with assumed unequal variance). Comparisons of any groups found to have a non‐Gaussian distribution were completed using Mann–Whitney non‐parametric analysis. The significance level for all tests was 0.05.

## 3 RESULTS

Tumour growth was well‐tolerated in all animals over the duration of study, with no premature decedent animals, nor clinical signs indicative of systemic toxicity/morbidity in any group. No abnormal macroscopic findings were present within any harvested endocrine tissues, there were no tumour‐ or treatment‐related group variations in body weight gain/adrenal weight, and the light microscopic appearances of the selected endocrine organs displayed no differences in H&E staining characteristics.

For vehicle control groups, preliminary analysis of 3D *Fibrelength Density* within the adrenal gland, pancreatic islets and thyroid interstitium, along with serological measurements of ACTH and TSH, indicated that the process of oral gavage versus i.p. vehicle dosing was unassociated with significant variation amongst these baseline values (Figure S2).

**FIGURE 1:**
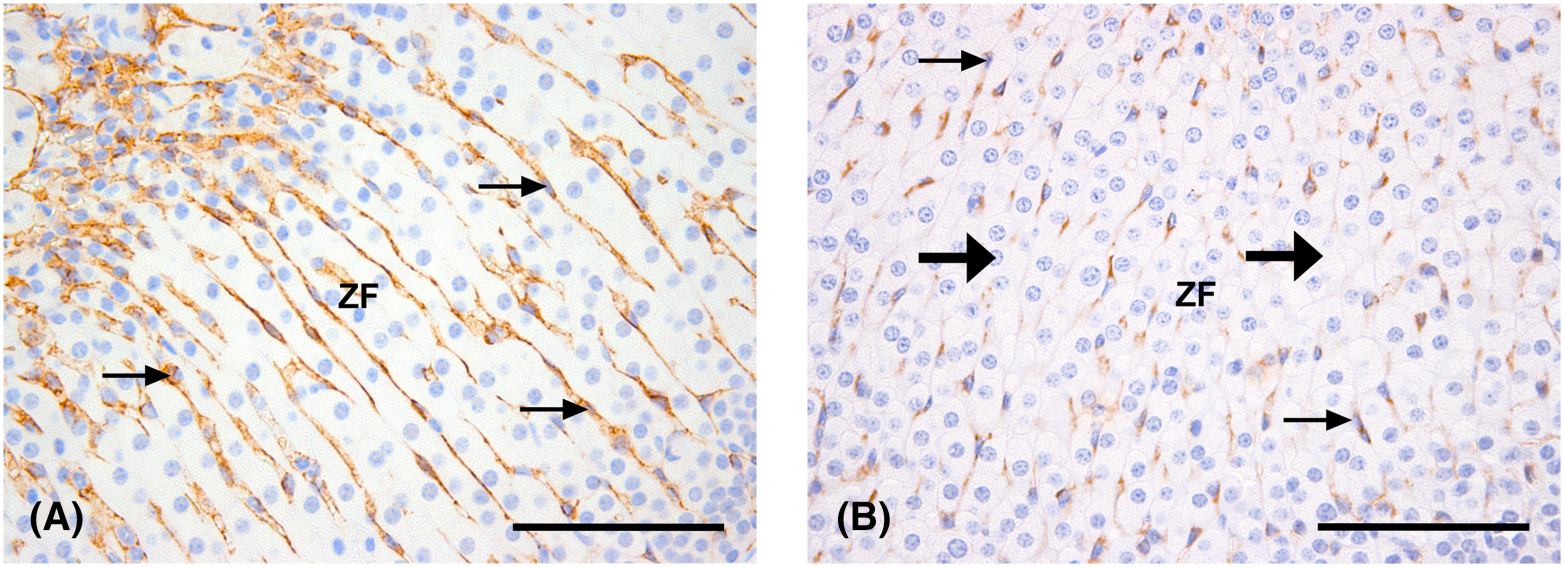
High‐power (original objective magnification 40×, scale bar = 100 μm) CD31‐stained sections of adrenal gland from a comparative vehicle control Calu‐6 tumour‐bearing mouse (A), and a Calu‐6 tumour‐bearing mouse administered high‐dose AZ10167514 (B). Note the apparent subjective decrease in cortical vascular profiles within the treated specimen. Vascular profiles are indicated with ‘thin’ arrows. ZF, region of the zona fasciculata. ‘Thick’ arrows in (B) indicate areas of cortical parenchyma devoid of significant vascular branching

With the application of CD31 IHC, via light microscopic evaluation, there were no visually subjective differences in pancreatic islet or thyroid interstitial vascularity between non‐tumour‐bearing and tumour‐bearing vehicle control mice. However, there appeared to be an increase in the density of vascular profiles, and in the extent of vascular (sinusoidal) branching/anastomoses, within the adrenal cortex, amongst a number of tumour‐bearing animals, when compared with non‐tumour‐bearing counterparts. This was similar to previous observations made when examining this tissue from xenograft‐implanted mice (Hargreaves et al., 2021). Animals administered high‐dose AZ10167514, in particular, also displayed a notable decrease in vascular (sinusoidal) branching/anastomoses within the adrenal cortex, in both non‐tumour‐bearing and tumour‐bearing individuals (Figure 1).

### 3.1 Structural endocrine vascularity

**FIGURE 2:**
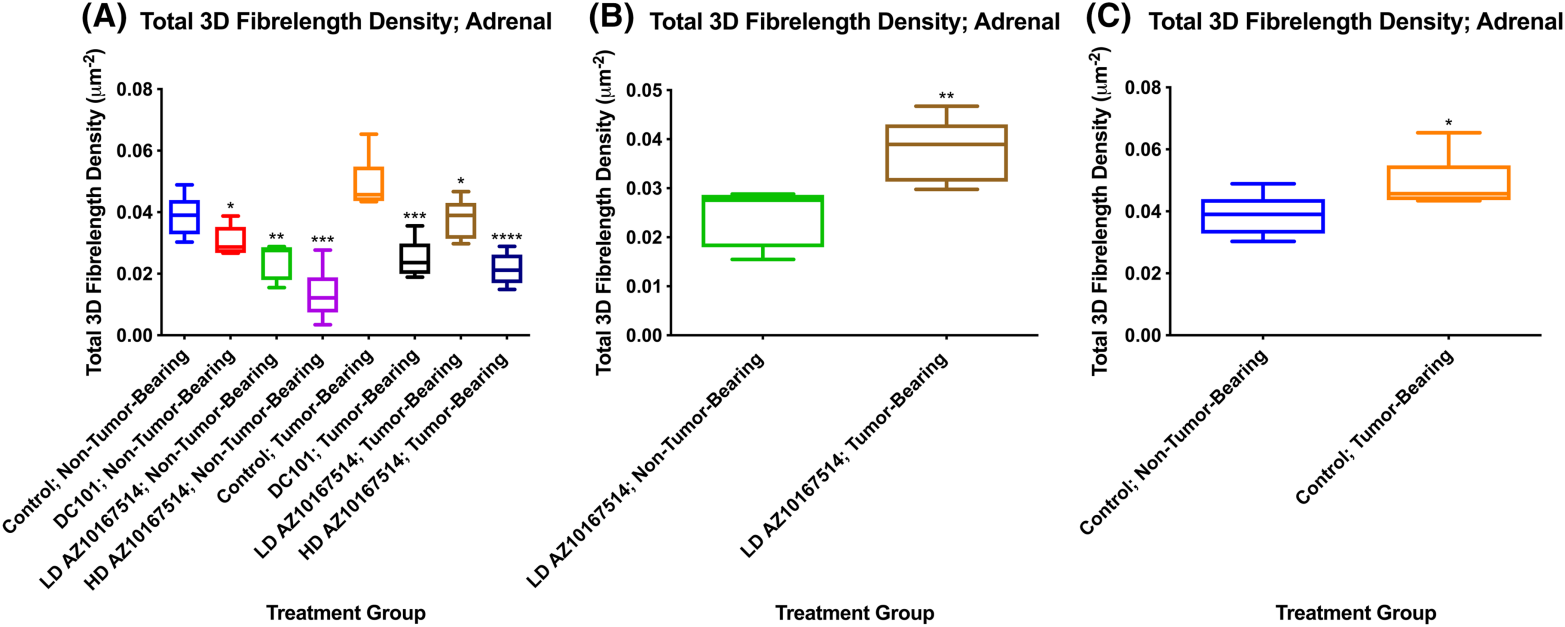
Plot showing that automated estimation of 3D *Fibrelength Density* demonstrated a decrease in vascular bed density within the adrenal gland of DC101 or AZ10167514 (6 or 12 mg/kg/day)‐treated non‐tumour‐bearing and tumour‐bearing mice, when compared with comparative vehicle control mice (A; oral gavage vehicle control group used for illustration). The extent of AZ10167514‐induced vascular rarefaction was attenuated, notably at the low‐dose, in tumour‐bearing animals (B). This effect was not present with DC101 administration. There was an apparent increase in vascular bed density within the adrenal gland of vehicle control Calu‐6 tumour‐bearing mice, when compared with non‐tumour‐bearing counterparts (C). LD, low‐dose (6 mg/kg/day); HD, high‐dose (12 mg/kg/day). Median +/− 10–90th percentile for 6 animals per group. *p < 0.05, **p < 0.01, ***p < 0.001, ****p < 0.0001

Automated analysis of 3D *Fibrelength Density* within the adrenal cortex revealed a number of findings (Figure 2).

When non‐tumour‐bearing comparative vehicle control and treated groups were contrasted, there was a statistically significant reduction in cortical vascularity amongst animals from all treatment groups. This appeared to be less marked in DC101‐administered animals (−39% group mean reduction) and most pronounced amongst high‐dose AZ10167514‐treated mice (−67% group mean reduction).

When tumour‐bearing comparative vehicle control and treated groups were contrasted, there was again a statistically significant reduction in adrenal cortical vascularity amongst animals from all treatment groups. However, when compared with non‐tumour‐bearing animals, the adrenal cortex retained higher vascular density measures for AZ10167514‐treated mice, notably at the low‐dose (+38% mean group increase in tumour‐bearing mice administered low‐dose AZ10167514, when compared with non‐tumour‐bearing mice administered low‐dose AZ10167514).

Finally, as previously described and also observed here (Hargreaves et al., 2021), there was an apparent increase in adrenal cortical vascularity amongst vehicle control Calu‐6 tumour‐bearing animals, when compared with the cohort of comparative vehicle control non‐tumour‐bearing animals.

Similar trends were present within the pancreatic islets and thyroid interstitium (Figure S3).

### 3.2 Endocrine status

With no Calu‐6 tumour burden, there was a relative increase in circulatory ACTH following low‐dose AZ10167514 and, to a lesser extent, DC101 administration. There were a number of observations in tumour‐bearing animals. Firstly, in vehicle control tumour‐bearing animals, there appeared to be a relative decrease in ACTH (and TSH) serum concentrations, when compared with non‐tumour‐bearing comparative vehicle control mice. In tumour‐bearing animals administered DC101 or AZ10167514 (at both doses), there was a comparative increase in ACTH release, potentially indicative of attempted cortical stimulation. At the high‐dose of AZ10167514, ACTH levels were of greater magnitude amongst tumour‐bearing, versus non‐tumour‐bearing, animals.

**FIGURE 3:**
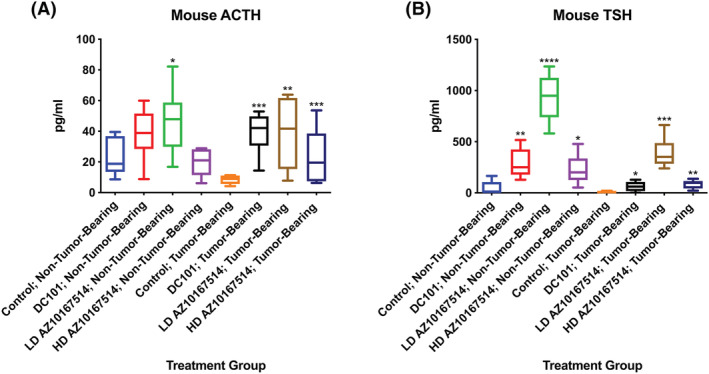
Plot showing multiplex serological probing for adrenocorticotrophic hormone (ACTH) (A) and thyroid stimulating hormone (TSH) (B) in non‐tumour‐bearing and Calu‐6 tumour‐bearing animals, with and without vascular modulating agent (VMA) administration. With no tumour burden, there was an apparent increase in the serum concentrations of ACTH with low‐dose AZ10167514 and, to a lesser extent, DC101 administration, when compared with comparative vehicle control groups. In tumour‐bearing animals given DC101 or AZ10167514 (at both doses), there appeared to be a comparative and consistent increase in circulatory ACTH. With or without a tumour burden, there was an increase in circulatory TSH amongst all treatment groups, notably with low‐dose AZ10167514 administration. Finally, in vehicle control tumour‐bearing animals, there appeared to be suppressed ACTH and TSH signalling, below that observed amongst non‐tumour‐bearing vehicle control counterparts (oral gavage control group used for illustration). LD, low‐dose (6 mg/kg/day); HD, high‐dose (12 mg/kg/day). Median +/− 10–90th percentile for 6 animals per group. *p < 0.05, **p < 0.01, ***p < 0.001, ****p < 0.0001

With no tumour burden, there additionally appeared to be relatively high serum levels of circulatory TSH amongst animals administered DC101 or AZ10167514 (at both doses). This was most marked in animals administered low‐dose AZ10167514. This trend was recapitulated in tumour‐bearing animals, albeit to a lesser degree (Figure 3).

### 3.3 Cytokine/growth factor signalling

**FIGURE 4:**
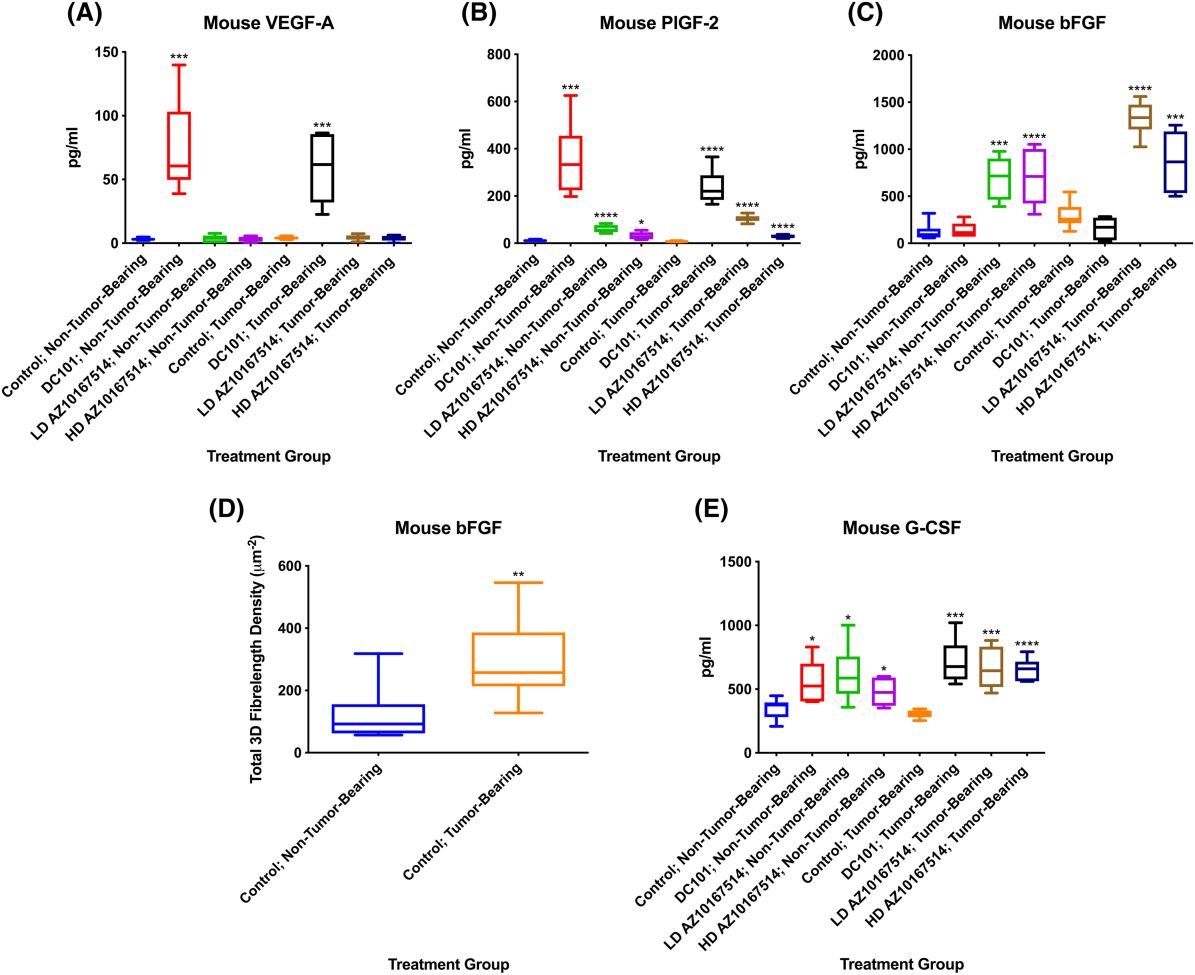
Plot showing multiplex serological probing for mouse‐specific vascular endothelial growth factor‐A (VEGF‐A) (A), placental growth factor‐2 (PlGF‐2) (B), basic fibroblast growth factor (bFGF) (C) and granulocyte colony stimulating factor (G‐CSF) (E) in non‐tumour‐bearing and tumour‐bearing animals, with and without vascular modulating agent (VMA) treatment, when compared with the oral gavage vehicle control group. With DC101 administration, there were increased serum concentrations of mouse‐specific VEGF‐A and PlGF‐2, the latter also apparent, to a lesser extent, with AZ10167514 administration. AZ10167514 administration was associated with increased serum levels of mouse‐specific bFGF, showing a greater elevation in tumour‐bearing animals, notably at the low dose. This assay also highlighted a differential concentration between vehicle control non‐tumour‐bearing and tumour‐bearing animals (D). With both DC101 and AZ10167514 administration, there was endogenous release of mouse‐specific G‐CSF. LD, low‐dose (6 mg/kg/day); HD, high‐dose (12 mg/kg/day). Median +/− 10–90th percentile for 6 animals per group. *p < 0.05, **p < 0.01, ***p < 0.001, ****p < 0.0001

With DC101 administration, there was endogenous release of mouse‐specific VEGF‐A, for both non‐tumour‐bearing and tumour‐bearing animals. There were also higher serum concentrations of mouse‐specific PlGF‐2; also apparent, albeit to a lesser extent, with AZ10167514 administration. With AZ10167514 administration, for both tumour‐bearing and non‐tumour‐bearing animals, there were further increases in the serum concentrations of mouse‐specific basic fibroblast growth factor (bFGF), showing a greater elevation in tumour‐bearing animals, notably at the low dose. This was not evident with DC101 administration. Additionally, the serum concentrations of mouse specific bFGF in vehicle control tumour‐bearing animals appeared higher, when compared with their vehicle control non‐tumour‐bearing comparative counterparts. Finally, with both DC101 and AZ10167514 administration, there were treatment‐related increased serum concentrations of mouse‐specific G‐CSF (Figure 4).

**FIGURE 5:**
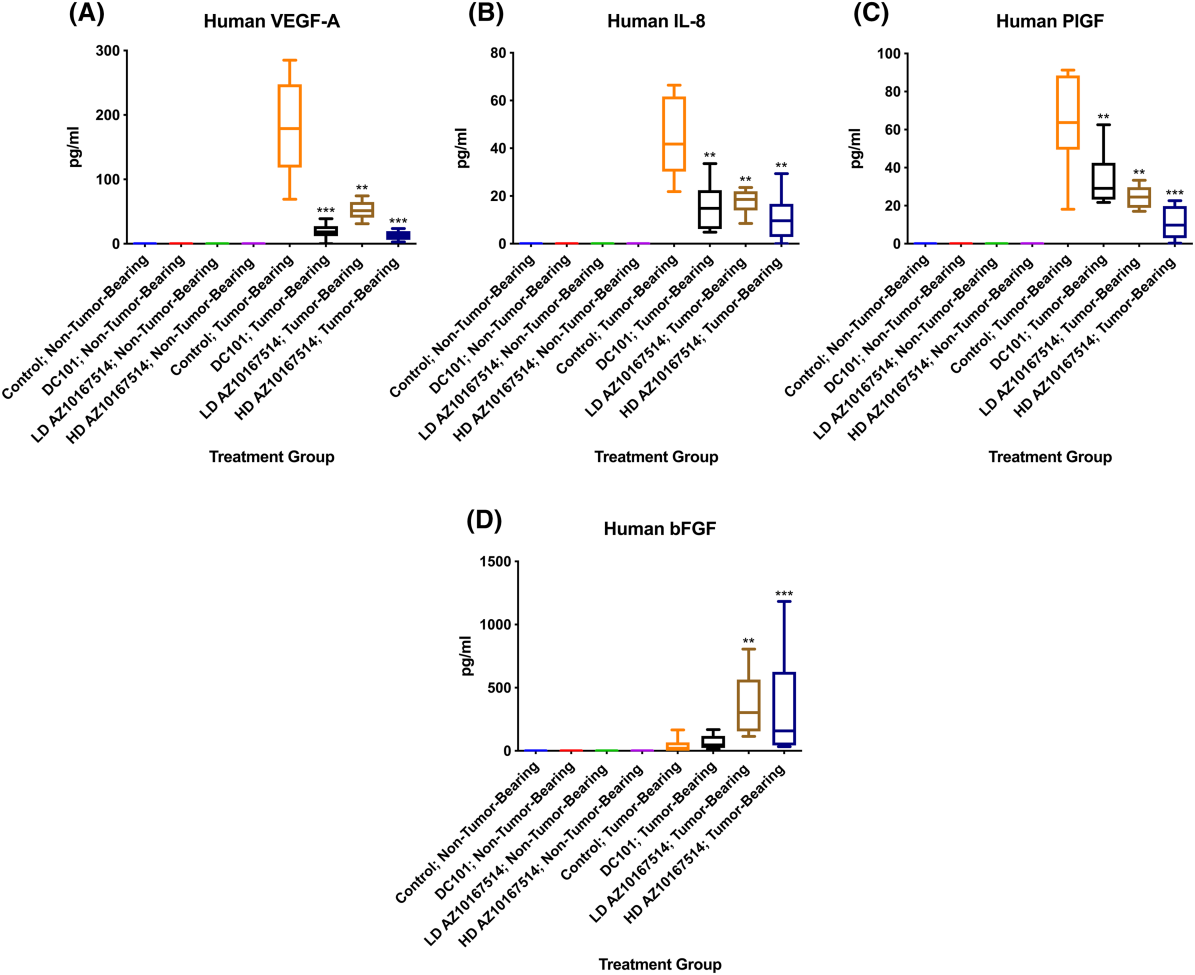
Plot showing multiplex serological probing for human‐specific vascular endothelial growth factor‐A (VEGF‐A) (A), interleukin‐8 (IL‐8) (B), placental growth factor (PlGF) (C) and basic fibroblast growth factor (bFGF) (D) in non‐tumour‐bearing and tumour‐bearing animals, with and without VMA treatment. In tumour‐bearing oral gavage vehicle control animals, there was a notable elevation in human‐specific VEGF‐A, IL‐8 and PlGF. This was suppressed with both DC101 and AZ10167514 administration. Amongst tumour‐bearing animals, there was a dose‐dependent increase in the measures of human‐specific bFGF with AZ10167514 administration. LD, low‐dose (6 mg/kg/day); HD, high‐dose (12 mg/kg/day). Median +/− 10–90th percentile for 6 animals per group. **p < 0.01, ***p < 0.001

In tumour‐bearing vehicle control animals, there appeared a marked serum elevation for human‐specific VEGF‐A, IL‐8 and PlGF. These elevations were substantially reduced with both DC101 and AZ10167514 administration; likely due to the reduced tumour mass following treatment. In contrast, amongst tumour‐bearing animals, there was a dose‐dependent increase in the release of human‐specific bFGF with AZ10167514 administration (Figure 5).

### 3.4 Western blot

**FIGURE 6:**
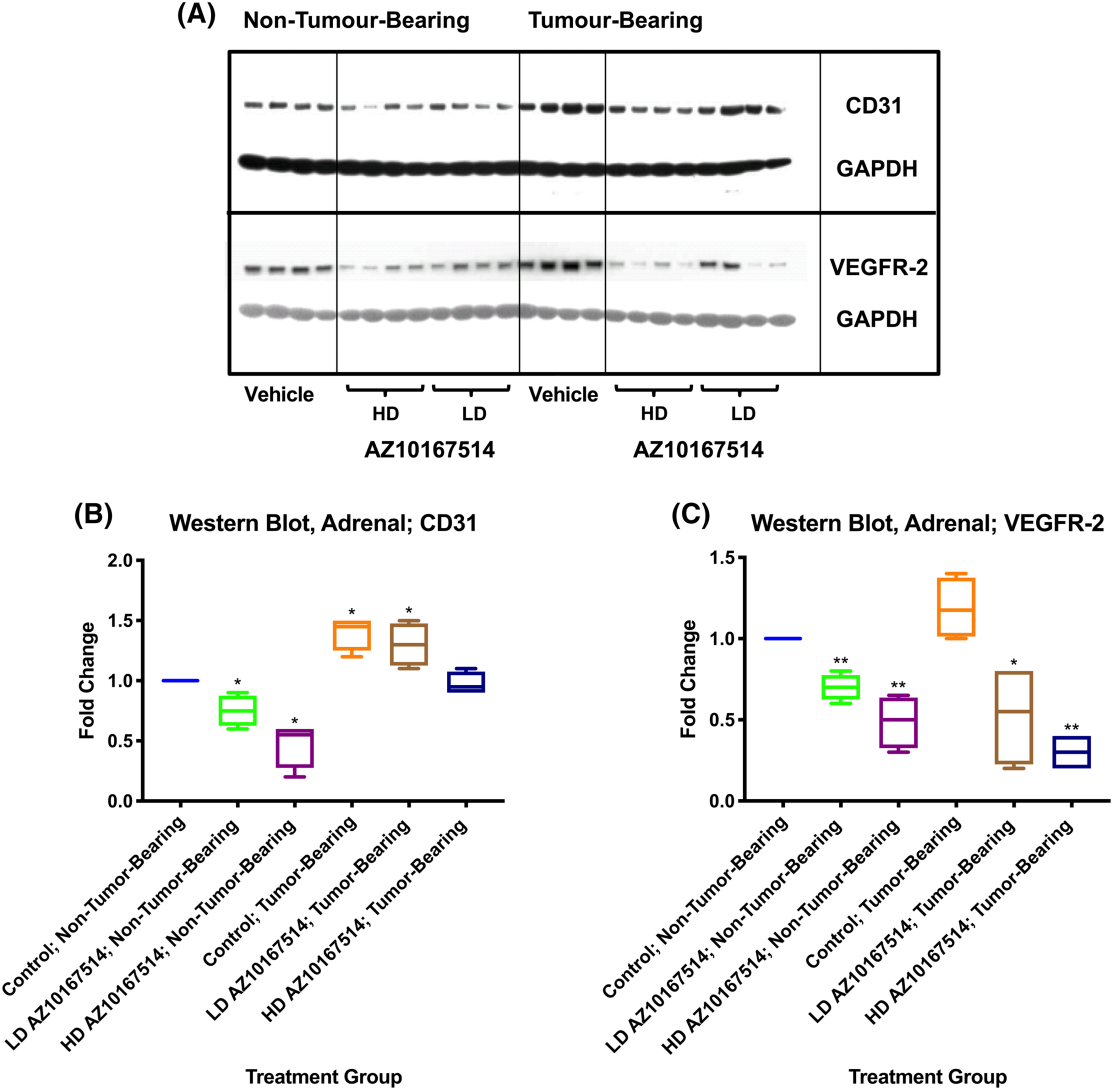
Gel blot (A) and data plots (B,C) displaying trends in adrenal gland CD31 (A,B) and vascular endothelial growth factor receptor‐2 (VEGFR‐2) (A,C) protein expression. Changes in CD31 expression were comparable with those observed from the measurements of 3D *Fibrelength Density* vascular analysis. These effects of tumour burden and treatment were not recapitulated in the levels of VEGFR‐2 protein expression. LD, low‐dose (6 mg/kg/day), HD; high‐dose (12 mg/kg/day). Median +/− 10–90th percentile for 6 animals per group. *p < 0.05, **p < 0.01

CD31 western blot analysis of the left adrenal gland from non‐tumour‐bearing and tumour‐bearing mice, in the presence or absence of AZ10167514 administration, mirrored the results obtained via 3D *Fibrelength Density* structural analysis. The dosing of 6 or 12 mg/kg, to non‐tumour‐bearing mice, led to a significant and dose‐dependent decrease in protein quantity. Likewise, in the presence of a Calu‐6 tumour burden alone, there was an apparent increase in CD31 quantity within this tissue, when compared with vehicle control non‐tumour‐bearing animals. On treatment of Calu‐6 tumour‐bearing animals with 12 mg/kg AZ10167514, there was a measurable reduction in adrenal gland CD31; however, this appeared of a lesser magnitude than that observed within non‐tumour‐bearing animals at this dose. At the dose of 6 mg/kg, CD31 expression was comparable with that observed in comparative vehicle control tumour‐bearing animals that had not been administered with the test article and at a level above that detected within comparative vehicle control non‐tumour‐bearing animals. VEGFR‐2 analysis indicated that this receptor was not comparatively upregulated, in terms of protein expression, amongst AZ10167514‐treated tumour‐bearing animals. There appeared a marginal increase in the quantity of this protein in vehicle control tumour‐bearing versus non‐tumour‐bearing animals, but this change did not attract statistical significance (Figure 6).

### 3.5 PCR

PCR analysis of Calu‐6 xenografts subject to VMA administration revealed that the dosing of 6 or 12 mg/kg AZ10167514 led to a significant increase in the expression of enpp2 (Autotaxin), when compared with the response generated amongst Calu‐6 tumours exposed to DC101.

Other genes with differential expression between DC101 and AZ10167514 treatment groups included apob (apolipoprotein B), apoc1 (apolipoprotein C1) and apoe (apolipoprotein E). These changes appeared more pronounced at 6 mg/kg AZ10167514 (Table 2).

**TABLE 2:** Genetic expression per comparative treatment group

Assay	Host mouse	Comparison group	Fold change	*p* value
Enpp2	LD AZ10167514	DC101	2.23	0.004
Enpp2	HD AZ10167514	DC101	2.56	0.004
Apob	LD AZ10167514	DC101	9.05	0.044
Aopc1	LD AZ10167514	DC101	6.43	0.003
Apoe	LD AZ10167514	DC101	6.24	0.046

Abbreviations: apob, apolipoprotein B; apoc1, apolipoprotein C1; apoe, apolipoprotein E; enpp2, autotaxin; HD, high‐dose (12 mg/kg/day); LD, low‐dose (6 mg/kg/day).

### 3.6 Discussion

We previously demonstrated that the implantation and growth of a range of mouse‐ and human‐derived allografts/xenografts leads not only to structural vascular changes within the peripheral host endocrine tissues but also imparts potential functional consequence. We noted that histogenetically diverse subtypes of tumour appeared stratified in their ability to influence peripheral endocrine vascularity and indicated a potential role in these observations for tumour‐ and host‐derived cytokines/growth factors, and the liberation of MDSCs (Hargreaves et al., 2021).

Preliminary investigations, undertaken during the development of novel structural biomarkers of vascular modulation (Hargreaves et al., 2017), had indicated that comparable ‘tumour–host interactions’ could additionally confer potential tolerance to the manifestation of anti‐angiogenic‐induced vascular rarefaction within the endocrine tissues of Calu‐6 tumour‐bearing mice (Figure S1).

With the use of this model of anaplastic lung carcinoma, here, we sought to establish a fuller understanding of the pathogenesis and potential consequence of these effects upon the induction of drug‐induced endocrine vascular injury.

Automated analysis of 3D *Fibrelength Density* within the adrenal cortex revealed a significant reduction in cortical vascularity amongst animals from all treatment groups, irrespective of tumour burden. When tumour‐bearing groups were contrasted, however, this effect was attenuated for AZ10167514‐treated mice, notably at the low‐dose. As described previously for other tumour types (Hargreaves et al., 2021), there was also an apparent increase in adrenal cortical vascularity amongst vehicle control Calu‐6 tumour‐bearing animals, when compared with the cohort of vehicle control non‐tumour‐bearing animals. This general trend was recapitulated within the pancreatic islets and thyroid interstitium.

Locally, intra‐tumoral resistance and adaptation to VMA/VEGF‐inhibiting anti‐angiogenics has been postulated to lead to variations in treatment response. For example, case studies have demonstrated examples of tumour relapse/regrowth, or increased progression, in glioblastoma patients following the cessation of bevacizumab in combination with chemotherapy dosing, and tumour ‘flare‐ups’ following the discontinuation of sunitinib or sorafenib therapy (Desar et al., 2009; Lu & Bergers, 2013). Such observations have been supported by the demonstration of increased tumour invasiveness and accelerated metastatic ‘conditioning’, amongst animal cancer models subject to anti‐VEGF administration (Ebos et al., 2009; Pàez‐Ribes et al., 2009). Potentially comparable events, involving vascular resistance/escape mechanisms, within host peripheral tissues, have not previously been reported.

In general, the administration of both AZ10167514 and DC101 was associated with a potential for increased adrenal and thyroid gland stimulation, as demonstrated by ACTH and TSH analysis. These changes generally appeared most prominent at the efficacious dose level for AZ10167514 and, in tumour‐bearing animals, attained greater significance for ACTH. This was seemingly due to lower baseline values in vehicle control tumour‐bearing mice. Levels of TSH in treated tumour‐bearing animals versus treated non‐tumour‐bearing mice were generally lower. Whether these trends in comparative TSH reduction were reflective of the lower baseline levels amongst tumour‐bearing animals, or representative of an increased capacity of this gland to function in the presence of a tumour burden, remains to be determined.

A number of physiologic and pathologic states have been associated with complex alterations in pituitary–adrenal/thyroid axis signalling, and increases in endocrine organ blood flow have been postulated as contributory (Boonen et al., 2013; Boonen et al., 2015; Kanczkowski et al., 2015; Kanczkowski et al., 2017; Lang et al., 1984; Peeters et al., 2015). Importantly, there is a potential for *stress* to contribute to endocrine signalling changes, primarily via increased ACTH/corticosterone release (Füchsl et al., 2013). Within the context of this study, the effects of both tumour growth and treatment could conceivably have contributed to a *stress* response. However, no group‐related changes in body weight gain were noted, no clinical signs consistent with morbidity were recorded, and the tumours appeared well‐tolerated throughout the duration of study. In addition, there were no tumour/treatment‐associated changes in adrenal weight, or light microscopic findings consistent with *stress* (e.g., cellular hypertrophy or alterations in zona fasciculata vacuolation), in any group. Furthermore, FACS analysis of the bone marrow did not reveal any findings consistent with a *stress* leukogram, the presence of a tumour burden in isolation was associated with a decrease in measured ACTH, and there did not appear to be a dose–response regarding ACTH liberation with AZD10167514 administration. These features gave weight to the conclusion that *stress* was unlikely to have materially influenced the observations reported (Everds et al., 2013).

The administration of a mixed TKI (AZ10167514) demonstrated that paraneoplastic resistance to VMA‐induced systemic endocrine vascular rarefaction may be most pronounced at comparatively lower dose levels (albeit at potentially clinically relevant exposures). Furthermore, despite a marked release of mouse‐specific VEGF‐A and PlGF‐2 with DC101 administration (features previously documented as compensatory test article‐related changes (Bocci et al., 2004), these findings did not appear to arise with VEGFR2 blockade in isolation. Western blot analysis of the adrenal gland showed that, whilst levels of CD31 expression appeared to confirm the structural vascular changes, as measured via 3D *Fibrelength Density* analysis, these findings were unaccompanied by comparable relative increases in VEGFR‐2 protein quantity, in tumour‐bearing animals subject to TKI administration. Human (xenograft)‐derived VEGF‐A, as measured in the serum, was also markedly attenuated with AZ10167514 administration. These observations suggested that the VEGF‐signalling cascade may be of limited importance within the findings reported here.

An analysis of human‐specific and xenograft‐derived cytokine/growth factors demonstrated significant circulatory concentrations of IL‐8 and PlGF (in addition to VEGF‐A), amongst tumour‐bearing animals. Whilst these mediators might also theoretically contribute to the observations of increased systemic endocrine vascularity, amongst tumour‐bearing mice, there appeared a general suppression, amongst all treatment groups, irrespective of the test item. These relative decreases were considered likely a result of decreased functional tumour mass/volume, following VMA administration (Table S1).

There was also a general drug‐induced release of host (mouse)‐derived G‐CSF, with all treatment protocols. However, changes were not significant between non‐tumour‐bearing and tumour‐bearing vehicle control mice. Additionally, an assessment of host bone marrow, via FACS analysis, failed to demonstrate significant shifts in the proportion of tumour‐associated myeloid progenitor/precursor lineages (in addition to CD202b‐positive EPCs), at this time point. It was therefore considered that this finding was likely a non‐specific and mild toxicologic effect of VMA administration.

The production of mouse‐specific bFGF was significantly increased with the administration of AZ10167514. Measured quantities were higher in treated tumour‐bearing animals, more notably at the lower dose. Additionally, there were increased concentrations in tumour‐bearing versus non‐tumour bearing vehicle control animals. Likewise, there was a marked release of human‐specific bFGF from the xenografts of AZ10167514‐administered animals, at both the low and high doses. These findings indicated that both host‐ and tumour‐derived bFGF may play a role in these observations of endocrine vascular rescue.

Amongst other mediators, the FGF signalling cascade has been proposed as one of a number of significant contributors to anti‐angiogenic treatment resistance (Itatani et al., 2018). Accordingly, treatment rational in overcoming FGF‐led resistance to anti‐VEGF therapy has centred around potential combinatorial approaches, with reference to the co‐administration of specific FGF inhibitors (Zahra et al., 2021).

FGFR1 is expressed on a number of cells, inclusive of endothelial and stromal/interstitial cells, both within the tumour and throughout the host (Poon et al., 2001). The upregulation of b‐FGF itself has been observed in tumours inherently resistance to anti‐VEGF therapy and notably those harbouring a local hypoxic environment (Casanovas et al., 2005; Yoshiji et al., 1997). It is liberated in tumour cell lines and tumours; both from the fibrovascular stroma and from the neoplastic cells themselves, the relative proportions of which may vary depending upon the specific histiogenic type, grade and stage (Chandler et al., 1999; Cronauer et al., 1997; Giri et al., 1999). It has more recently been postulated that the activation of the FGF signalling pathway, specifically within the tumour vasculature itself, may be the more important pathway in the development of anti‐VEGF‐acquired resistance (Ichikawa et al., 2020).

The paracrine action of bFGF, in stimulating local tumour‐associated angiogenesis via FGFR1, may be accompanied by rising levels of the growth factor within the sera of affected patients, the measurement of which has been used as a prognostic biomarker (Akl et al., 2016). Within the context of this evaluation, it was apparent that bFGF liberation from the human‐derived xenograft cells themselves was accompanied by significant bFGF secretion from host tissues, both appearing to increase with AZ10167514 administration. Given the production of host‐derived bFGF in non‐tumour‐bearing animals, it was apparent that potential sources included tissues distant to the (mouse‐derived) intra‐tumoural stroma itself. Further investigation would elucidate the potential for FGF‐mediated effects upon the endocrine vasculature directly, although a possible link between therapeutic FGFR inhibition and the development of hypothyroidism, potentially as a consequence of direct vascular disruption, has been proposed (Ahn et al., 2019).

A concomitant finding to these observations was the demonstration, via PCR, of enhanced enpp2 (Autotaxin) expression amongst Calu‐6 tumours exposed to AZ10167514, over and above that observed following the dosing of DC101. The role of autotaxin, in both angiogenesis and in the systemic stabilization of blood vessels, has been documented, in addition to the ability of bFGF to upregulate this factor (Igarashi et al., 2021; Nam et al., 2000; Rogers et al., 2004). Furthermore, the autotaxin‐lysophosphatic acid signalling axis has been associated with the development of acquired resistance to the administration of sunitinib in renal carcinoma (Su et al., 2013). This is postulated to be a result of intra‐tumoural hypoxia, with consequent circumvention of the established VEGF‐VEGFR‐mediated angiogenic pathway (Huang et al., 2010; Quan et al., 2017; Zhang et al., 2021). It has also recently been documented that the autotaxin‐lysophosphatic acid signalling cascade may closely associate with lipoprotein transport and metabolism in cancer inflammation and progression (Benesch et al., 2018; Brindley et al., 2020). We additionally noted within this study that there appeared to be shifts in intra‐tumoural lipoprotein ratio expression, notably at the 6 mg/kg AZ10167514 dose.

Additional mechanistic investigation is warranted to further our understanding into the off‐target effects of VMA agents within host endocrine tissues; how these may be similar in aetiology to findings noted within tumours; how the tumour and host may interact in modulating these sequelae; and the precise pathogenesis of disrupted endocrine physiology, in both cancer and VMA treatment settings.

Here, we have highlighted that tumour‐bearing mice may manifest differing degrees of systemic vascular toxicity when administered VMAs. The adrenal gland, in particular, has also been shown to act as a robust sentinel tissue, by which to evaluate these off‐target effects. These findings suggest that the dosing of new and emerging anti‐angiogenic therapies, to healthy preclinical rodent species, may enhance the degree of drug‐induced systemic vascular toxicity, in a non‐diseased setting, and that susceptible target tissues could be evaluated, by which to assess the specificity of novel and more targeted drug candidates.

## 4 CONCLUSION

Here, we demonstrate that the implantation and growth of the Calu‐6 xenograft are associated with a resistance to VMA‐induced host endocrine tissue vascular rarefaction (toxicity), with potential functional impact and notably with respect to mixed TKI. The pathogenesis of these findings indicates a potential role for both tumour‐ and host‐derived bFGF, with associated upregulation in the intra‐tumoural autotaxin‐lysophosphatic acid signalling axis.

Further understanding of preclinical tumour–host interactions, and their potential to alleviate or exacerbate the off‐target effects of anti‐angiogenic (and other drug) treatments, could aid in the prediction of therapeutic efficacy versus potential adverse clinical outcome, in a defined cancer population.

## CONFLICT OF INTEREST

The authors have no conflicts of interest to declare.

## WEBSITE REFERENCES

Merck Millipore (n.d.). PROTOCOL_FOR_00003798MAN_MPTMAG‐49K; PROTOCOL_HAGP1MAG‐12K; PROTCOL_MAGPMAG‐24K. Retrieved from: https://www.merckmillipore.com/GB/en/product/MILLIPLEX-MAP-Mouse-Pituitary-Magnetic-Bead-Panel-Endocrine-Multiplex-Assay,MM_NF-MPTMAG-49K; https://www.merckmillipore.com/GB/en/product/MILLIPLEX-MAP-Human-Angiogenesis-Growth-Factor-Magnetic-Bead-Panel-Cancer-Multiplex-Assay,MM_NF-HAGP1MAG-12K; https://www.merckmillipore.com/GB/en/product/MILLIPLEX-MAP-Mouse-Angiogenesis-Growth-Factor-Magnetic-Bead-Panel-Cancer-Multiplex-Assay,MM_NF-MAGPMAG-24K.

## Supporting information


**Table S1:** Mean tumor volume, standard deviation, and p value per vehicle control/treatment group at the start and end of study. i.p.; Intra‐peritoneal, LD; Low‐dose (6 mg/kg/day), HD; High‐dose (12 mg/kg/day), ns; not significant.

**Figure S1:** Plot showing that automated estimation of 3D *Fibrelength Density* detected an attenuated decrease in vascular bed density within the adrenal gland (A) and thyroid gland (B) of M396647‐treated Calu‐6 tumor‐bearing mice, from a previous study, when compared to treated non‐tumor‐bearing counterparts. There was no comparable trend noted for pancreatic islet vascularity (C). Median +/− 10‐90th percentile for 6 animals per group. *p < 0.05.

**Figure S2:** Plot showing that automated estimation of 3D *Fibrelength Density* within the adrenal gland (A), pancreatic islets (B), and thyroid gland (C), along with multiplex serological probing for ACTH (D) and TSH (E), in non‐tumor‐bearing and Calu‐6 tumor‐bearing vehicle control animals, did not detect a statistically‐significant variation within these baseline measures, between oral gavage vehicle and i.p. vehicle administration. The effects of DC101 administration upon these end‐points incorporated the i.p. vehicle control values for statistical comparison (Figures 2 and 3; Supplementary Figure 3).

**Figure S3:** Plots demonstrating that automated estimation of 3D *Fibrelength Density* detected comparable trends in non‐tumor‐bearing versus tumor‐bearing mouse pancreatic islet (A) and thyroid interstitial (B) vascularity, as documented for the adrenal gland (Figure 2; oral gavage vehicle control group used for illustration). LD; Low‐dose (6 mg/kg/day), HD; High‐dose (12 mg/kg/day). Median +/− 10‐90th percentile for 6 animals per group. * *p* < 0.05, **p < 0.01, ***p < 0.001, ****p < 0.0001.

## Data Availability

Data available on request from the authors.
