# Supplementary material for: Tumours modulate the systemic vascular response to anti‐angiogenic therapy
Source: J Appl Toxicol. 2022 Mar 2;42(8):1371–84. doi: 10.1002/jat.4301 (PMC9543901; doi:10.1002/jat.4301)
Supplement: Supplementary file 1 — Table S1: Mean tumor volume, standard deviation, and p value per vehicle control/treatment group at the start and end of study. i.p.; Intra‐peritoneal, LD; Low‐dose (6 mg/kg/day), HD; High‐dose (12 mg/kg/day), ns; not significant. [file JAT-42-1371-s003.docx]

| **Treatment Group:** | **Mean Tumor Volume; Start (cm^3^):** | **Standard Deviation:** | **p value:** | **Mean Tumor Volume; Final (cm^3^):** | **Standard Deviation:** | **p value:** |
| --- | --- | --- | --- | --- | --- | --- |
| **Vehicle Control (Oral)** | **0.46** | **0.12** | **-** | **1.34** | **0.35** | **-** |
| **Vehicle Control (i.p.)** | **0.44** | **0.16** | **ns** | **1.28** | **0.43** | **ns** |
| **DC101** | **0.41** | **0.18** | **ns** | **0.64** | **0.24** | **0.0031** |
| **LD AZ10167514** | **0.40** | **0.17** | **ns** | **0.73** | **0.15** | **0.0060** |
| **HD AZ10167514** | **0.47** | **0.12** | **ns** | **0.37** | **0.06** | **0.0009** |

**Supplementary Table 1: Mean tumor volume, standard deviation, and p value per vehicle control/treatment group at the start and end of study. i.p.; Intra-peritoneal, LD; Low-dose (6 mg/kg/day), HD; High-dose (12 mg/kg/day), ns; not significant.**
