# Supplementary figures and images for: Tumours modulate the systemic vascular response to anti‐angiogenic therapy
Source: J Appl Toxicol. 2022 Mar 2;42(8):1371–84. doi: 10.1002/jat.4301 (PMC9543901; doi:10.1002/jat.4301)

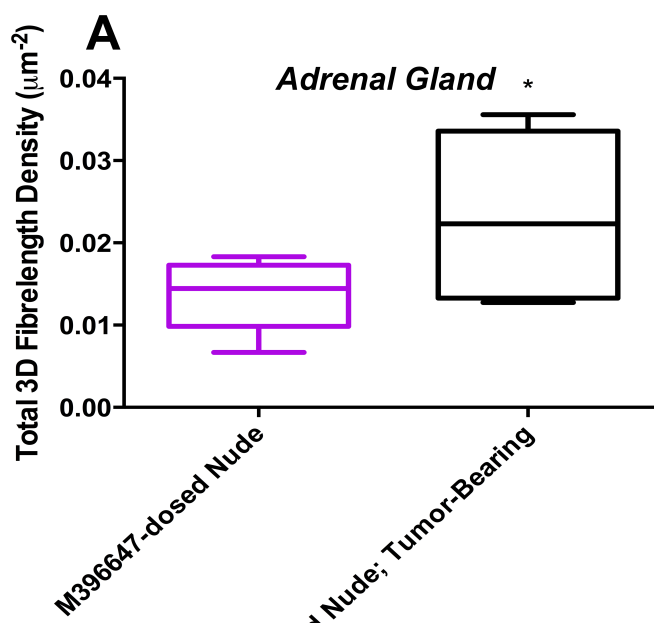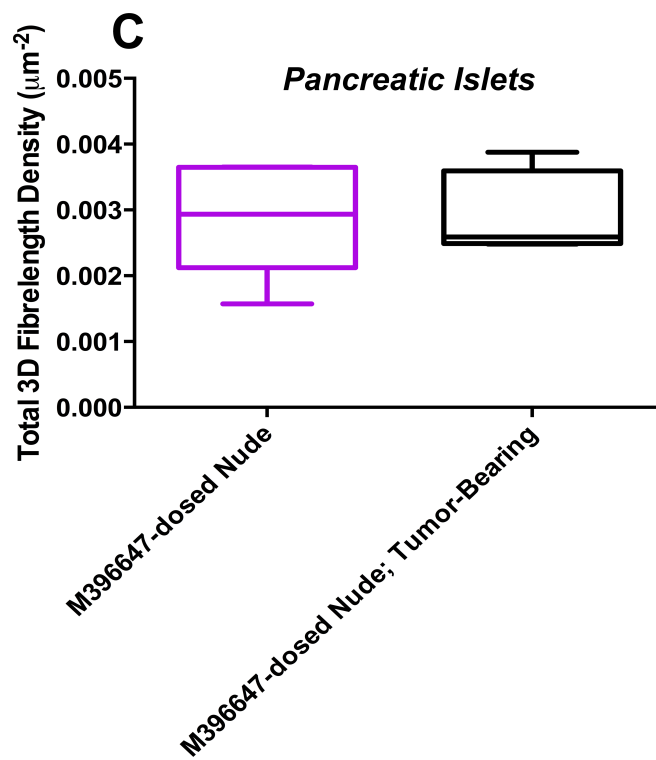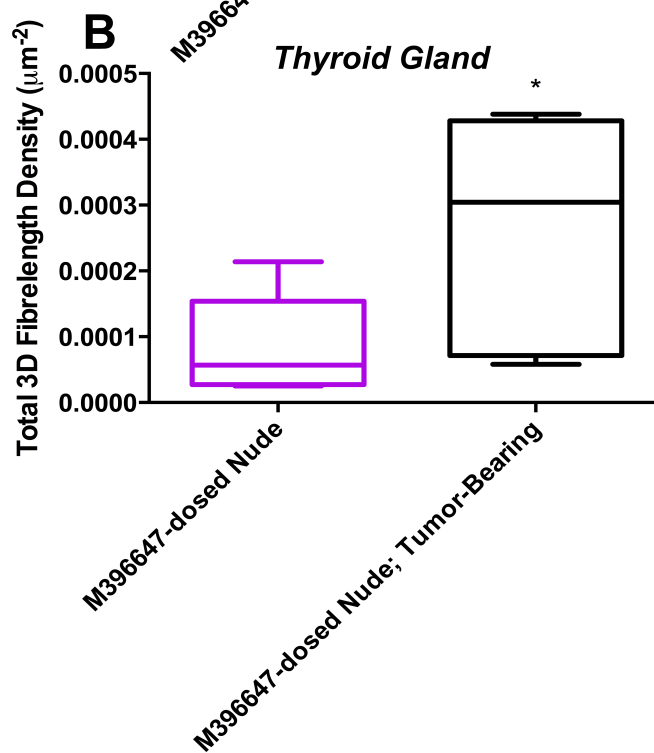

Supplement: Supplementary file 2 — Figure S1: Plot showing that automated estimation of 3D Fibrelength Density detected an attenuated decrease in vascular bed density within the adrenal gland (A) and thyroid gland (B) of M396647‐treated Calu‐6 tumor‐bearing mice, from a previous study, when compared to treated non‐tumor‐bearing counterparts. There was no comparable trend noted for pancreatic islet vascularity (C). Median +/− 10‐90th percentile for 6 animals per group. *p < 0.05. [file JAT-42-1371-s002.pdf]

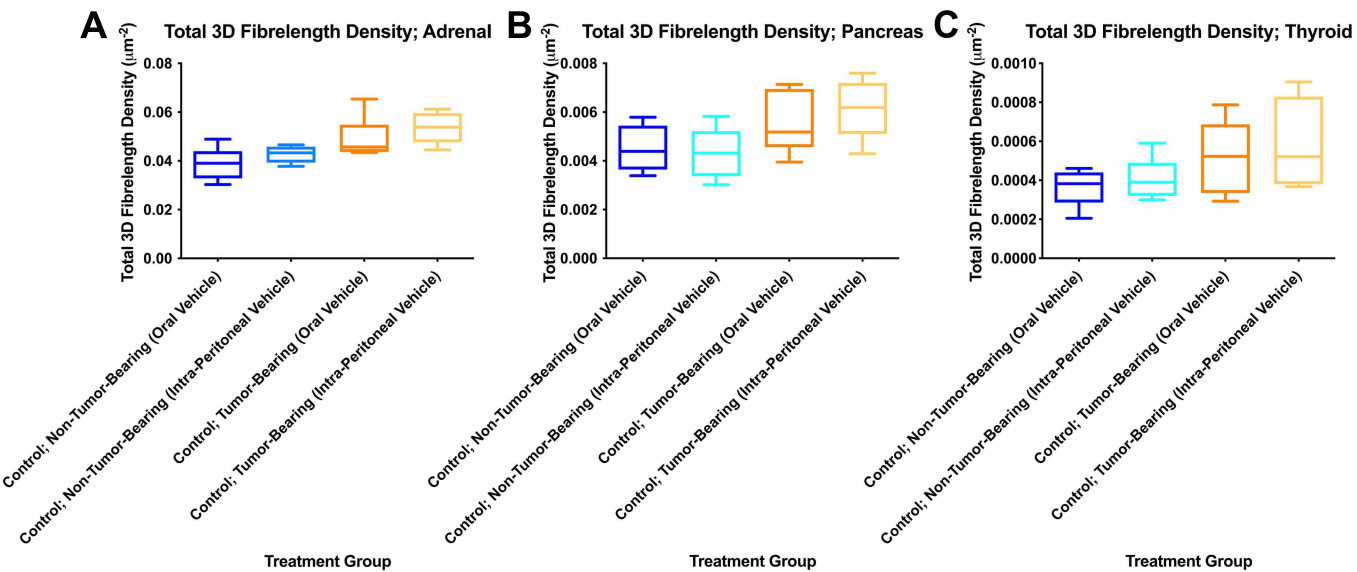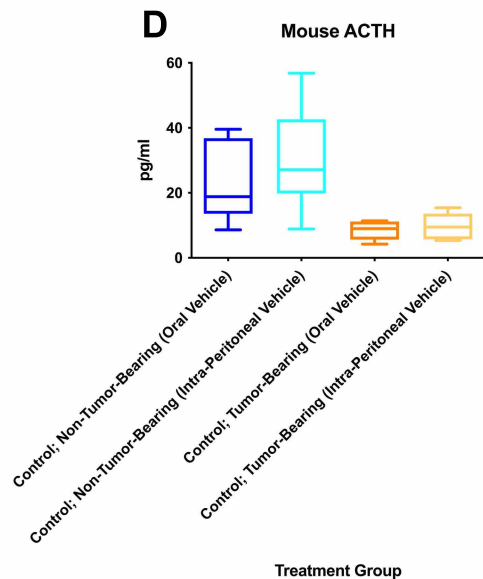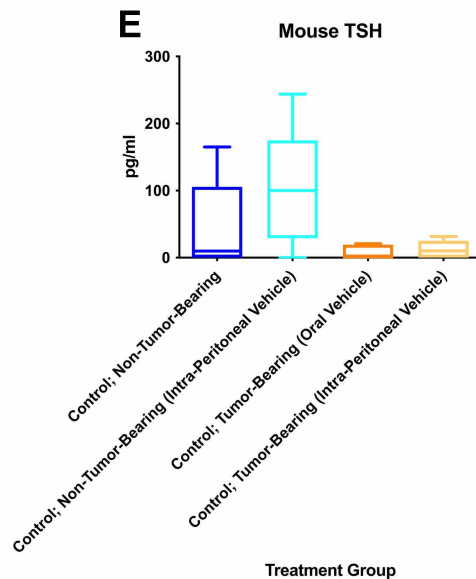

Supplement: Supplementary file 3 — Figure S2: Plot showing that automated estimation of 3D Fibrelength Density within the adrenal gland (A), pancreatic islets (B), and thyroid gland (C), along with multiplex serological probing for ACTH (D) and TSH (E), in non‐tumor‐bearing and Calu‐6 tumor‐bearing vehicle control animals, did not detect a statistically‐significant variation within these baseline measures, between oral gavage vehicle and i.p. vehicle administration. The effects of DC101 administration upon these end‐points incorporated the i.p. vehicle control values for statistical comparison (Figures 2 and 3; Supplementary Figure 3). [file JAT-42-1371-s004.pdf]

**A** Total 3D Fibrelength Density; Pancreas

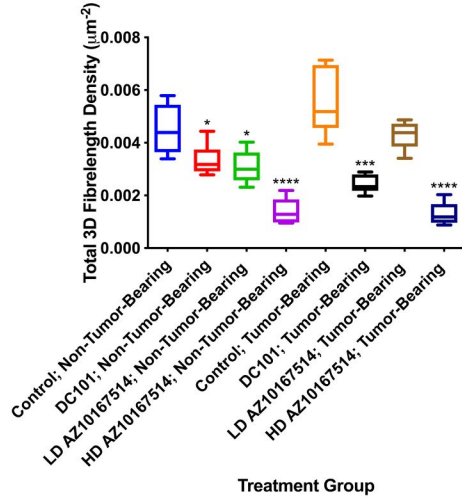

**B** Total 3D Fibrelength Density; Thyroid

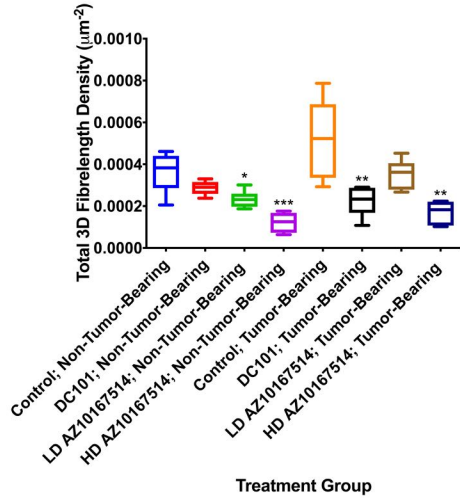

Supplement: Supplementary file 4 — Figure S3: Plots demonstrating that automated estimation of 3D Fibrelength Density detected comparable trends in non‐tumor‐bearing versus tumor‐bearing mouse pancreatic islet (A) and thyroid interstitial (B) vascularity, as documented for the adrenal gland (Figure 2; oral gavage vehicle control group used for illustration). LD; Low‐dose (6 mg/kg/day), HD; High‐dose (12 mg/kg/day). Median +/− 10‐90th percentile for 6 animals per group. * p < 0.05, **p < 0.01, ***p < 0.001, ****p < 0.0001. [file JAT-42-1371-s001.pdf]
